# Working Towards Inclusive, Socially Accountable and Resilient Community Health Systems: An Introduction to a Special Issue

**DOI:** 10.34172/ijhpm.2021.166

**Published:** 2021-12-13

**Authors:** Charles Michelo, Anna-Karin Hurtig, Helen Schneider

**Affiliations:** ^1^School of Public Health, University of Zambia, Lusaka, Zambia.; ^2^Department of Epidemiology and Global Health, Umeå University, Umeå, Sweden.; ^3^School of Public Health and SAMRC Health Services to Systems Research Unit, University of the Western Cape, Cape Town, South Africa.

**Keywords:** Primary Healthcare, Resilience, Trust, Community Health Systems

## Abstract

This editorial introduces the eleven papers in the special issue titled: *The multiple lenses on the community health system: implications for research and action*. Our editorial begins by describing the collaboration that led to the special issue, and then gives an overview of the contents of the special issue, which include two framing papers and nine empirical contributions from researchers in Zambia, Tanzania, Sweden, South Africa, India, and Australia. We conclude by considering how these papers collectively speak to the theme of resilience.

## Background

The 40th anniversary of the Alma Ata Declaration on primary healthcare (PHC), in the context of the Sustainable Development Goals, has provoked a re-valuing of the idea of ‘community’ in health systems.^1,2^ In the World Health Organization’s (WHO’s) recent PHC Operational Framework, ‘empowered people and communities’ are one of three pillars of PHC, alongside ‘multisectoral policy and action’ and ‘integrated health services.’^3^ Most would agree that these foundational elements of PHC – in particular the first two - remain aspirations rather than realities in most health systems. Nevertheless, over the last years, the pendulum has swung firmly in favour of ‘community health’ – typically bringing together ideas of community participation and community-based service provision – and increasingly becoming the subject of formal policies and programmes.

Mindful of this global context, this special issue on the theme of community health systems (CHS) has been co-edited in a collaboration between health policy and system researchers from the Universities of Zambia, Western Cape (South Africa), and Umeå (Sweden). In mid-2019 we hosted a gathering at the Chaminuka Lodge in Lusaka, Zambia, where over one week, we followed a structured process of pooling our collective insights on the elements and purposes of CHS. The result was a typology of four key ‘lenses’ on the CHS, articulated as ‘programmatic,’ ‘relational,’ ‘collective action’ and ‘critical’ lenses, representing different positionalities and purposes in the CHS. We also developed a set of research priorities and compiled a joint ‘Chaminuka Manifesto on the Community Health System,’ outlining a set of common values and ethical stances for research and practice in the CHS.^4^

 There are eleven papers in the issue, two of which are short communications authored by the Chaminuka collective, followed by nine empirical contributions from Zambia, Tanzania, Sweden, South Africa, India, and Australia.

This special issue complements other recent efforts offering new thinking on community health.^5-7^ It is distinctive in being conceived and authored by embedded researchers close to country policy and implementation processes. To use Seye Abimbola’s framing,^8^ the papers have a local ‘pose,’ and they address themes peculiar to specific contexts, with a ‘gaze’ that could also be considered ‘local.’ Why then would we seek to collate these case studies in an international journal, ie, for a foreign audience? The answer to this lies firstly, in the relevance of complex, ‘organic’ as opposed to specific ‘surgical’ knowledge^8^ on the CHS for lesson learning across jurisdictions. Thick descriptions from one context may provoke others in similar or even different settings to think afresh about their own realities; they may also widen the scope of what to study in the CHS, beyond well-established instrumental foci such as maternal-child health programmes. The papers touch on a diverse range of themes, for example, community dynamics in rural health worker retention, the politics of the policy process, collective action in times of crisis and mechanisms of responsiveness. Secondly, the papers, when seen together, represent a natural way in which system complexity must be examined; the knowledge generated from such multifaceted viewpoints can be considered a *public health good* applicable in both local and global social and geographical scales.

## A Synopsis of the Papers

The first paper which frames the series is titled *The Multiple Lenses on the Community Health System: implications for policy, practice, and research,* and elaborates the four key lenses and their implications for policy, practice and research. These lenses reflect the multidisciplinary collaboration underpinning this special issue and capture something of the terrain that is the CHS. We do not seek to elevate any of the lenses as more relevant or valid than another, but rather propose the necessity of holding them simultaneously for meaningful action on the CHS, even if the lenses appear to be opposites. The second short communication reports on the structured process adopted to arrive at the research priorities for the CHS and the manifesto.

The nine papers which follow the two framing papers represent the four lenses. Several papers are written through a *relational lens*, showing the salience of research on social dynamics within the CHS. Two explicitly foreground the role of trust in shaping performance – Zulu and colleagues^9^ explore how prioritising trust relations unlocked access to care for the stigmatized condition of hydrocoele in Zambia, while Assegaai and Schneider^10^ conversely describe how wider institutional mistrust generated low interpersonal trust between frontline actors in a community health worker programme. Mathias et al^11^ conducted participatory action research which surfaced unique local knowledge for the design of mental health care in Uttarakhand State, India. Finally, Sirili et al^12^ offer novel insights into rural health worker retention in Tanzania by locating the problem as one of relationships with the community - either accommodation or rejection – rather than the usual focus on health system (supply side) incentives.

Two papers illustrate the *critical lens* on the CHS. Drawing on the experience of Australia, Baum and Freeman^13^ analyse the political economy, namely the ideas, interests, and institutions, of why CHS do not flourish in high income countries. They point to the power of biomedicine and a retreat from investment in public PHC under neoliberalism. Another of the contributions from the Zambian collective illustrates how a confusing array of (mostly international) actors and interests behind the development of a community health strategy crowded out frontline provider and community perspectives.

In a case study of *collective action*, van Ryneveld and colleagues^14^ write about the mobilization of community action networks across Cape Town in response to the livelihood and social crisis precipitated by the harsh lockdowns at the start of the coronavirus disease 2019 (COVID-19) pandemic. They reprise the central role of trust relations in enabling a rapid mobilization of the community action networks, which they refer to as “moving at the speed of trust.” The authors also describe the difficulties of a fluid and self-organised social movement attempting to engage formalised state responses, an indication of the gap between the easy rhetoric of social mobilization and its reality in practice.

Finally, two of the papers, from Sweden and South Africa, address the design and implementation of strategies in the CHS at a more macro level, and are in part *programmatic* and in part relational in their lenses. Using a realist methodology, Jonsson and colleagues^15^ unpack the mechanisms at play in the shift from innovator sites to scale up of ‘virtual health rooms’ in communities of rural northern Sweden, providing insights into the necessary conditions for such a transition that include, amongst others, relationships of trust. Sutherns and Olivier^16^ map the mechanisms for receiving and responding to citizen feedback – complaints, hotlines, facility committees and satisfaction surveys, amongst others – in the public health system of one South African province, concluding on the need for far greater synergy if these mechanisms are to amount to meaningful ‘health system responsiveness.’

## Resilience, Trust and the Community Health Systems

Ultimately, the intention of these papers is to appreciate the role of the CHS more fully within health systems and to understand how to nurture its functioning. Our starting point is to acknowledge CHS functioning as an inherently contested idea, with many viewpoints, explanations, and reflections. Listening to the ‘voices’ of the papers in this collection, however, we would argue that the processes that lead to such functioning can be referred to as a kind of *CHS resilience,* and the drivers that shape or worsen resilience is the knowledge these papers bring.

Resilience here is understood as a response to both sudden shocks, such as COVID-19,^17^ as well as chronic stressors^18^; and crucially, the capacity to go beyond absorption or (mal)adaptation to one of transformation, able to challenge the causes of shocks and stressors.^18^ As several of the papers illustrate, such ‘process resilience’ requires ways of functioning, including leadership, coordinated priority setting, empowered individuals with agency able to operate flexibly, communities that work together, inclusion and participation and effective planning and coordination around the common goal of responding to people’s health needs.^19,20^ The key output of process resilience is trust, a central theme in the papers, whether in the participatory design of mental health care in Uttarakhand, community mobilisation in South Africa, integration of virtual health rooms in Sweden or the uptake of hydrocoele treatment in Zambia. CHS resilience is nurtured through community actions and actors that enhance trust.

More broadly, we think that interpreting CHS functioning as a combination of processes, outputs and outcomes of resilience adds a new dimension to the debate of what has not worked and why in PHC; what could work; and what can inform policy and practice as a necessary prerequisite to realising the Alma Ata Declaration on PHC.^21^ We would go further to assert that without strengthening CHS resilience, universal health coverage will be unattainable.^18^

The system wide and potentially transformative role of the CHS is, however, far from being recognised and even less achieved. In most settings, the CHS is moribund, treated as a subordinate sphere within health system hierarchies, with inequitable status and poor connections to the overall health system, and little deliberate investment.^22^ This has been most vividly demonstrated in the ravages of COVID-19, especially in contexts where community engagement and trust relations are weak and the CHS is poorly integrated into national health systems.^23^ Building a resilient CHS remains a monumental challenge. It encompasses layers of complexity, as systems must establish links from national governments to households, from the public to the private sphere, and from health to other sectors.^6,18^

 To achieve CHS resilience, the interface between community health and the overall health system must be enabled by deliberate acts, underpinned by an information base, governance that addresses power asymmetries, local finance systems and effective health delivery. However, our investments should be multi-pronged, requiring not just hardware, but also the software of relationships which emanate from appropriate community engagement, empowerment, mutually accountable partnerships, and equitable processes led by trusted governors.

## Acknowledgements

 We are deeply indebted to the IJHPM editorial team for so readily embracing the idea of this special issue. The journal plays a uniquely interesting role, not least of which is that it publishes papers from across the global North and South, and has an epistemic inclusivity that mirrors what we believe is required in policy, practice and research on CHS.

## Ethical issues

 Not applicable.

## Competing interests

 Authors declare that they have no competing interests.

## Authors’ contributions

 CM, HS, AKH jointly conceived the editorial; CM and HS drafted the editorial; all approved the final version.

## Funding

 South African National Research Foundation, Swedish STINT and Swedish Research Council.

## Authors’ affiliations

^1^School of Public Health, University of Zambia, Lusaka, Zambia. ^2^Department of Epidemiology and Global Health, Umeå University, Umeå, Sweden. ^3^School of Public Health and SAMRC Health Services to Systems Research Unit, University of the Western Cape, Cape Town, South Africa.
